# Rush Medical College Clinic

**Published:** 1877-09

**Authors:** Edmund M. Landis


### RUSH MEDICAL COLLEGE CLINIC.

### Service of Prof. Moses Gunn.

(Reported by Edmund M. Landis, M. D.)

*Lateral Curvature.*

C. F., German, aged 15, is a poorly nourished lad, whose appetite is good, but who eats sparingly of fats. He states that he was well up to four months ago, when he first noticed pain in his right side, which began to be crooked, and that his breathing was embarrassed.

On examination, find a lateral curvature to the right side, and a prominence in the dorsal vertebral region. On stooping, at request, to pick up an article from the floor, he bends over with ease and freedom. This little experiment at once dismisses the suspicion of disease of the vertebræ, and we have lateral curvature resulting in a corresponding angle at the back, causing the suspicious prominence noticed at first. The respiratory movement of the chest seems good, yet the patient complains that he cannot breathe well. On making pressure on the chest, find that we can materially restore its natural shape.

The question, in this case, is whether we shall advise the usual apparatus, or suspension by the arms and hands from a swing. [See a paper on suspension as a means of treatment in Spinal Distortions, by Benjamin Lee, M. D., of Philadelphia, read before the last meeting of the American Medical Association, by Dr. Frank Woodward, of Philadelphia.] We have a case under treatment for whom a modification of both apparatus and swing is being used. The patient had double lateral curvature, and the bowing in on the left side was so great that the chest could almost be spanned with the fingers. The respiration of the left side was much embarrassed. The only means of treatment had been a stiff corset, which tightly compressed the chest. An apparatus was made for this patient, the essential features of which were a pelvic band or girth, a part which corresponded to the natural curve of the spine, and armlets or crutches to support the arms, and to keep the shoulders back. This made no pressure on the chest. With this, the child was put in the swing and made to frequently inspire and expire as fully as she could. Full diet was ordered.

That child has steadily improved, the curvature is being reduced, and the embarrassed breathing relieved.

In this case we would hardly recommend an apparatus, as a swing would be all sufficient.

*Epithelioma.*

J. B. W., aged 58, German, farmer. The patient, whose previous health had been good, states that he first noticed, some two or three years ago, in the right side of the mouth, close to the gums, a small whitish looking, painless ulcer, which he thought to be a simple sore. This not disappearing he consulted a physician, who treated it for sometime without success, and finally cauterized it. After this the sore began to spread, ulcerate and pain,—the pain being of a sharp, shooting character. He had several teeth extracted, hoping by their removal to gain some benefit. The sore continued to enlarge and spread. Three months ago he consulted a physician, who pronounced the disease carcinoma.

On examination, find an infiltration in the right cheek, extending within and without, covering an area of about two inches, and approaching the angle of the lips. It is fixed, immovable and ulcerated internally and externally. The gums about the disease, and also on the opposite side of the jaw, present a number of whitish looking deposits, or patches. There is some pus, but no ichorous discharge. The alveolar process is involved, and we find it softened, broken down, and filled with sinuses, the disease also spreading to the soft parts on the inside of the mouth. On probing with several probes in different directions, find that one of them passes clear through the cheek, and alveolar process to the side of the tongue.

We have here an example of epithelioma a little out of the general type. Its advent, progress and present appearance, are singular. It began in the duplicature of the mucous membrane just where it begins to be reflected upon the gums, commencing, as is now observed, with a thickened condition of the mucous membrane, as a number of patches of a snowy white appearance. Epithelioma does not usually begin in the mucous membrane as a white degeneration. Seen at the first glance, one would call it a diphtheritic deposit, but on examination find it a part of the mucous membrane. The patient complains of a soreness which is characteristic of pus, and pus is found, but the ichorous discharge which we would expect is absent. The characteristic pain is present, and the disease is apparently spreading to the opposite side.

If an operation were made it would be necessary to remove the whole of that part of the jaw involved in the disease, and we have examined the case with reference to this, and find that a section made through the symphisis menti, and another as far back as the first molar teeth, would accomplish the object.

The large amount of the soft parts of the cheek which would be involved in the operation would make a gape, that would be difficult to close. But even if we were successful in this, on account of the liability of a disease to return, which has progressed so far and attained to such a degree as this, we cannot conscientiously recommend the operation.

We inferred that in the first stage of this disease the whitened condition of the mucous membrane was singular. This is not the case in the later stages. Remember having removed an epulis, where the patient came back after a few weeks with a return of the disease, when this same whitened thickened condition of the mucous membrane was present.

